# Comprehensive Transcriptome Analysis of Rare *Carpinus putoensis* Plants under NO_2_ stress

**DOI:** 10.3390/genes12050754

**Published:** 2021-05-17

**Authors:** Qianqian Sheng, Congzhe Liu, Min Song, Jingyuan Xu, Zunling Zhu

**Affiliations:** 1 College of Landscape Architecture, Nanjing Forestry University, Nanjing 210037, China; qqs@njfu.edu.cn (Q.S.); Jeremy@njfu.edu.cn (C.L.); songmin@njfu.edu.cn (M.S.); xujingyuan@njfu.edu.cn (J.X.); 2 Co-Innovation Center for Sustainable Forestry in Southern China, Nanjing 210037, China; 3 College of Art & Design, Nanjing Forestry University, Nanjing 210037, China

**Keywords:** transcriptome, NO_2_ stress, high-throughput sequencing, molecular mechanism, resistance, gene expression

## Abstract

We evaluated a transcriptome using high-throughput Illumina HiSeq sequencing and related it to the morphology, leaf anatomy, and physiological parameters of *Carpinus putoensis putoensis* under NO_2_ stress. The molecular mechanism of the *C. putoensis* NO_2_ stress response was evaluated using sequencing data. NO_2_ stress adversely affected the morphology, leaf anatomy, and total peroxidase (POD) activity. Through RNA-seq analysis, we used NCBI to compare the transcripts with nine databases and obtained their functional annotations. We annotated up to 2255 million clean Illumina paired-end RNA-seq reads, and 250,200 unigene sequences were assembled based on the resulting transcriptome data. More than 89% of the *C. putoensis* transcripts were functionally annotated. Under NO_2_ stress, 1119 genes were upregulated and 1240 were downregulated. According to the KEGG pathway and GO analyses, photosynthesis, chloroplasts, plastids, and the stimulus response are related to NO_2_ stress. Additionally, NO_2_ stress changed the expression of POD families, and the *HPL2*, *HPL1*, and *POD* genes exhibited high expression. The transcriptome analysis of *C. putoensis* leaves under NO_2_ stress supplies a reference for studying the molecular mechanism of *C. putoensis* resistance to NO_2_ stress. The given transcriptome data represent a valuable resource for studies on plant genes, which will contribute towards genome annotations during future genome projects.

## 1. Introduction

Nitrogen dioxide (NO_2_) is a product of nitric acid, which is used in industrial manufacturing; millions of tons of NO_2_ are produced each year [1]. At high temperatures, NO_2_ is a maroon gas with a typically harsh odor, and it is a key contributor to air pollution [2]. NO_2_ is also an important component of acid rain [3]. Its corrosivity and highly oxidative nature make it harmful to plant biochemical and physiological processes after entering plants through the stomata [4]. In wild environments, the ambient NO_2_ level that wild plants might encounter is 180 ppb. Currently, there are two theories regarding the effect of NO_2_ on plants. The first is that NO_2_ can form plant organic nitrogen compounds by being metabolized and amalgamated in the nitrate assimilation pathway [5]. Approximately 33% of NO_2_-derived N (NO_2_-N) taken up by plants was modified into a previously unknown Kjeldahl-unrecoverable organic nitrogen (unidentified nitrogen) [6], which can be incorporated into the α-amino group of soluble free amino acids [7,8], thereby not causing harm to the leaves [9,10]. Mayer et al. [11] investigated the changes in the physiological functions of NO_2_ at a 10 µL L^−1^ concentration in *Arabidopsis* (*Arabidopsis thaliana*) cells and found that 1 h NO_2_ fumigation induced pathogen resistance in the plant [11]. The second theory is that the majority of plants have a low absorption capacity for NO_2_-N incorporation into the total plant N and can resist NO_2_ [12]. Although most studies have investigated the amino acid response after NO_2_ stress, there are no known reports on gene expression responses to NO_2_ stress.

*Carpinus putoensis* is a species in the *Betulaceae* family measuring approximately 15 m (49 feet) tall. It survives as a single tree on Putuo Island on the Zhoushan archipelago in China. It is monoecious but still able to reproduce sexually in nature [13]. The Zhejiang Forestry Science Research Institute has researched the cultivation and breeding of *C. putoensis* [14]; although the seed characteristics of *C. putoensis* were investigated previously, those studies stressed the characterization of the complete *chloroplast genome* and nuclear ribosomal sequence data [15]. It is vital to study *C. putoensis* resistance to NO_2_ exposure to conserve this endangered species and improve its tolerance for future applications as a novel road greening and ornamental plant. Therefore, in a previous study, we evaluated the photosynthesis and *Chl* fluorescence responses of *C. putoensis* leaves to different NO_2_ (6 μL/L) exposure times, both in terms of leaf gas exchange and the functionality of photosynthetic measurements [16]. Additionally, the chlorophyll content, the behavior of the stomata, and the ultrastructure of chloroplasts were analyzed together to find potential relationships between the photosynthesis in the leaves and cell transformation under NO_2_ stress. However, a relationship between the leaf anatomy and transcription in *C. putoensis* under NO_2_ stress has not previously been reported.

Therefore, in the current research, we evaluated the leaf anatomy and transcriptome gene expression of *C. putoensis* leaves under NO_2_ stress. The purpose of this study is to provide a theoretical reference on the effects of traffic pollution on green plants.

## 2. Materials and Methods

### 2.1. Plant Material and Growth Conditions

One-year-old *C. putoensis* seedlings were grown in pots measuring 30 cm (open top) × 15 cm (height) × 20 cm (flat bottom) that were filled with well-mixed vermiculite, peat, and garden soil (1:1:1, *v*/*v*/*v*). In accordance with the water evaporation rate of the soil described by Allen et al. [17], they were watered with tap water every three days, and 1 L of full-strength Hoagland nutrient solution at was used biweekly for seedling cultivation. Before NO_2_ treatment, the plants were allowed to grow naturally for 2 months [16]. 

### 2.2. NO_2_ Fumigation

Fumigation was performed according to the method described in the literature [11]. open-top NO_2_ fumigation glass chambers measuring 50 × 50 × 50 cm were built. The plants were fumigated with NO_2_ at 6 μL/L that was supplied by cylinders (gas flow velocity, 1 L/min). The *C. putoensis* seedlings in another climate chamber constituted the control (CK) group, which was quantitatively flushed with filtered air (without NO_2_) at the same time. The chambers underwent a light/dark cycle with a photoperiod of 13 h and had a relative humidity of 60/50 ± 4% (day/night), with a temperature of 25/20 ± 3 °C (day/night). The control and NO_2_-treated seedlings (30 replicates in each treatment) were fumigated for 3 days (6 h per day), and then they recovered for 30 days [16].

The NO_2_ concentration within the climate chamber containing leaves exposed to 1 L/min of air was measured with an NO_2_ analyzer (model ML Series). After being treated with NO_2_, the seedlings were placed in an artificially controlled greenhouse under a natural simulation environment for 30 days of recovery. The environmental conditions of the greenhouse were as follows: room temperature, 25–28 °C; relative humidity, 60–70%; photoperiod, 14 h; and photosynthetically active radiation, 1000 μmol photons/(m^2^ s).

For the following experiments, whole leaves were used unless otherwise specified. 

### 2.3. Determination of Total Peroxidase (POD) Activity

POD is a class I oxidation-reduction enzyme that acts as a catalyst in a variety of biological processes; thus, it is an essential protective enzyme against reactive oxygen cell damage [18]. In response to adversity, POD is activated and provides resistance against adverse oxidation stress [19]. In this study, the POD level was measured with a guaiacol colorimeter [20]. The samples were pooled, and approximately 0.2 g of fresh leaves was placed in a pre-chilled mortar and then ground with 0.2 g of quartz sand. A total of 6 mL of 0.05 mol/L phosphate buffer (pH, 7.5) was added (in three applications, including one for mortar rinsing). The resulting homogenate was poured into a 10 mL centrifuge tube and stored at 4 °C. Centrifugation was performed at 5000× *g* for 20 min, and the obtained supernatant was a crude extract of POD. The reaction system for measuring the enzymatic activity contained 2.9 mL of phosphate buffer (0.05 mol/L), 1.0 mL of H_2_O_2_ (2%), 1.0 mL of guaiacol (0.05 mol/L), and 0.1 mL of enzymatic solution. The enzymatic solution was boiled for 5 min and used as the control. After the enzymatic solution was applied, the system was immediately subjected to a 15-min incubation at 37 °C, which was followed by an ice bath. Trichloroacetic acid (20%, 2.0 mL) was added to terminate the reaction. Filtration (Steripak-GP, 10 L; Millipore, Germany) and appropriate dilution were then performed. The absorbance was measured at 470 nm [20]. Six replicates were designed for each group.

### 2.4. Transmission Electron Microscopy (TEM)

The plant material was cut into 1-mm^2^ pieces and then fixed with 2.5% glutaraldehyde in a 0.1 M sodium cacodylate buffer (pH 7.4) for 4 h. After three washes with cacodylate buffer, the samples were fixed in 2% (*w*/*v*) osmium tetroxide in cacodylate buffer for 2 h. The samples were embedded in epoxy resin and dehydrated with an acetone series. Sections were cut using an LKB III ultramicrotome at 1 μm for light microscopy (LM) and approximately 50 nm for TEM. Ultrathin sections were stained with uranyl acetate and basic lead citrate and then analyzed by a Hitachi Hu 12a electron microscope [16].

### 2.5. RNA Isolation, cDNA Library Construction, and Illumina Sequencing

To understand the changes in gene levels after NO_2_ fumigation, we selected the CK group and the 72-h NO_2_ treatment group for transcriptome sequencing analysis. Two groups were prepared: a NO_2_ treatment group and a CK group. After the leaves were removed from the tree, they were pooled and immediately frozen in liquid nitrogen and then stored at −80 °C in an ultra-low temperature freezer. The total RNA was extracted using the cetyltrimethy lammonium bromide (CTAB) method [21] and treated with RNase-free DNase I (TaKaRa, Dalian, China). The total RNA integrity was checked using gel electrophoresis, and the content was quantified using an ND-1000 spectrophotometer (Thermo, Waltham, MA, USA). Oligo (dT) 25 magnetic beads were used for isolating poly-(A) tail-containing mRNAs from the total RNA (20 μg), and mRNA was disrupted into short fragments with a fragmentation buffer at 70 °C for 5 min. These short fragments were used as templates to synthesize first-strand cDNA using random hexamer primers and reverse transcriptase. Second-strand cDNA fragments were obtained using a buffer containing DNA polymerase I, dNTPs, and RNase H. The final cDNA library was obtained by ligating the cDNA fragments to sequencing adaptors (Genomic DNA Sample Preparation Kit, Illumina, San Diego, CA, USA; two terminal sequencing: read length, 150 bp; paired end) and by conducting PCR amplification (Illumina Genomic Sample Preparation Kit, Illumina, San Diego, CA, USA). An Illumina HiSeq 2000 platform (Macrogen Bioinformatics Technology, Shenzhen, China) was used to sequence the mRNAs. Three replicates were designed for each group. 

### 2.6. Data Analysis for RNA-seq Experiments

Adaptor sequences and low-quality reads were removed from the raw reads to obtain clean data [22,23]. The trinity method was adopted to assemble the clean data into transcripts [24]. National Center for Biotechnology Information, U.S. National Library of Medicine (NCBI) BLAST was used to compare the transcripts with NR, Swiss-Prot, Gene Ontology (GO), euKaryotic Orthologous Groups (KOG), Kyoto Encyclopedia of Genes and Genomes (KEGG), and several PFAM databases to obtain functional annotations [25]. The procedures for the RNAseq sequencing evaluation were as follows: Bowtie2 was used to compare the effective data from the samples to the spliced transcripts, and the mapping information was counted; Rseqc was used to analyze the redundant sequences and the distribution of inserted fragments; and BEDtools was used to check the homogeneity distribution and analyze the gene coverage [26]. A gene structure analysis was then performed. Specifically, BCFtools was used to seek possible SNP sites according to the mapping results; MISA was used for SSR analysis based on the sequence information of the spliced transcripts [27]. Salmon was used to calculate the gene expression. WGCNA was used for gene co-expression analysis. Based on the expression matrix of the samples, multi-directional statistical analyses and exploration, such as comparative analyses of the samples, were performed [28,29].

### 2.7. Identification, Annotation, and Enrichment Analysis of Differentially Expressed Genes

To identify *differentially expressed genes* (DEGs) related to the leaf metabolism of *C. putoensis* after NO_2_ stress, we used RNA-seq by expectation maximization (RSEM) to map the clean reads of each sample to the transcriptome assemblies, and we used the DESeq with the following thresholds for DEG identification: false discovery rate (FDR), 0.01; fold change, 2 [30]. The identified DEGs were then used for GO and KOG classification and KEGG pathway enrichment analysis.

### 2.8. Validation by RT-qPCR

The results from the RNA-seq experiment were validated by analyzing eight plant genes that were most significantly differentially regulated under NO_2_ stress (the smallest *p*-value was 1 × 10^−30^ for chloroplasts) using RT-qPCR with cDNA as the template. RNA was obtained using the same method described in the Section 2.5. Oligo 7 software was used to design all the primers for RT-qPCR (Supplementary Table S1). A TB Green Premix Ex Taq kit (TaKaRa, Shiga, Japan) was used to perform RT-qPCR and an ABI StepOne plus thermal cycler (Applied Biosystems, Foster City, CA, USA) was used to run the RT-qPCR.

## 3. Results

### 3.1. Morphology and Cell Structure of C. putoensis Leaves 

**Figure 1:**
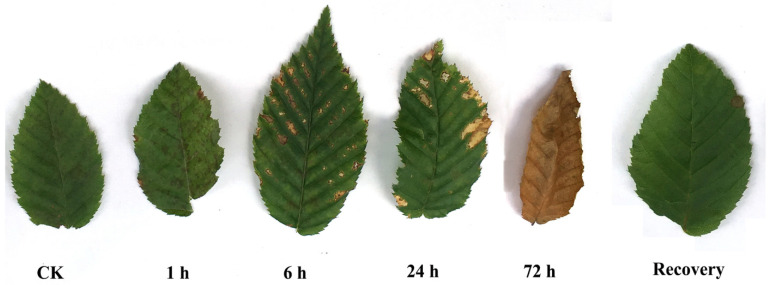
Morphological changes in *C. putoensis* leaves receiving NO_2_ treatment. CK: control group. NO_2_ treated: 1 h, 6 h, 24 h, 72 h, and recovery for 30 days.

**Figure 2:**
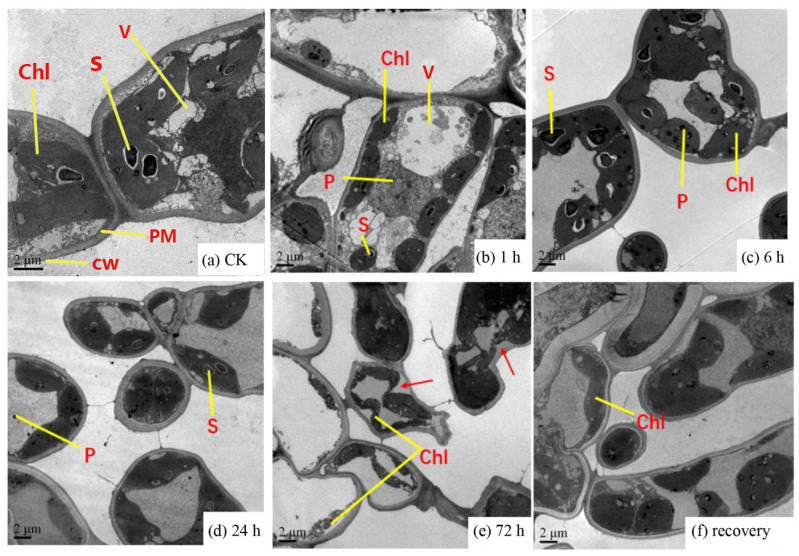
Images of cell structures from the primary leaf under a transmission electron microscope. (**a**), control; (**b**–**e**), NO_2_-treated plants; and (**f**), recovery plants. From 1 h to 72 h, the plastoglobuli in the cells gradually increase, and the chloroplasts gradually shrink, and they become slender and sticky. Slowly, the cell wall is separated. The red arrows in (**e**) indicate plasmolysis. V, vacuole; P, plastoglobuli; PM, plasma membrane; S, starch grain; CW, cell wall; and Chl, chloroplasts.

The leaf morphology exhibited various changes when *C. putoensis* was exposed to NO_2_ gas. According to Figure 1, the *C. putoensis* leaf damage appeared mostly as necrotic spots, from black spots to yellow spots, to an increasing extent. Some areas (such as the leaf tip) were severely damaged under NO_2_ stress for 1–72 h. Figure 2 shows the micrographs of leaves from the control group and the treated plants after 1 h, 6 h, 24 h, and 72 h of NO_2_ treatment under a TEM. The CK group exhibited oval cells with numerous well-compartmentalized grana stacks within Figure 2a. Minimal starch was present. In NO_2_-treated plants (Figure 2b,c), most cell structures were slightly damaged. Within some cells, cell dehydration and shrinkage (Figure 2d) and chloroplast deformation (Figure 2e) were found. Plastoglobuli were more numerous in the disrupted cells. The chloroplasts of the recovery plant leaves are discoidal and rich in starch grains (Figure 2f). Following recovery from the NO_2_ treatment, almost all the cell structures and chloroplasts in the plants seemed to recover their normal morphology. Starch was present in all cases. In some chloroplasts, the thylakoid system was almost unaffected. Few differences were observed between the plastids of the CK group and those of the NO_2_-treated plants that had recovered for 72 h (Figure 2a,f).

### 3.2. Changes in POD Activity

**Figure 3:**
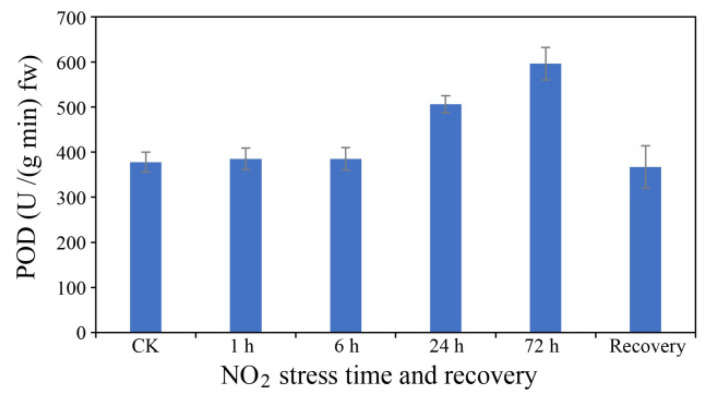
POD activity after different NO_2_ stress times or 30 days of recovery. Six replicates for each group.

Changes in the POD activity of *C. putoensis* at different NO_2_ stress time points are shown in Figure 3. With increasing NO_2_ fumigation time, the POD activity of *C. putoensis* increased, ranging from 385 U/(g min) fw to 596 U/(g min) fw. The 72-h treatment group had the highest POD level, with a significant difference compared to any of the remaining groups. Compared with the CK group, the 24 h treatment group showed a significant difference. The recovery group did not show a significant difference from the CK group.

### 3.3. RNA-seq Analysis of Clean Data from C. putoensis

*C. putoensis* is a non-model organism; therefore, de novo assembly is the only option for sequence assembly. In de novo assemblies, without the guidance of a reference sequence, the reads are assembled into contigs. To cover the *C. putoensis* transcripts completely, de novo assembly was used to generate the consensus transcriptome using Illumina sequencing data from samples under two different conditions together with raw reads from NO_2_-treated leaves and CK leaves. Due to trimming (extra bases whose lengths were lower than 20) and duplicate removal, we analyzed 529,540 transcripts with an average length of 425.97 bp for the de novo assembly of 250,200 unigenes with an average length of 376.73 bp (Table 1). 

**Figure 4:**
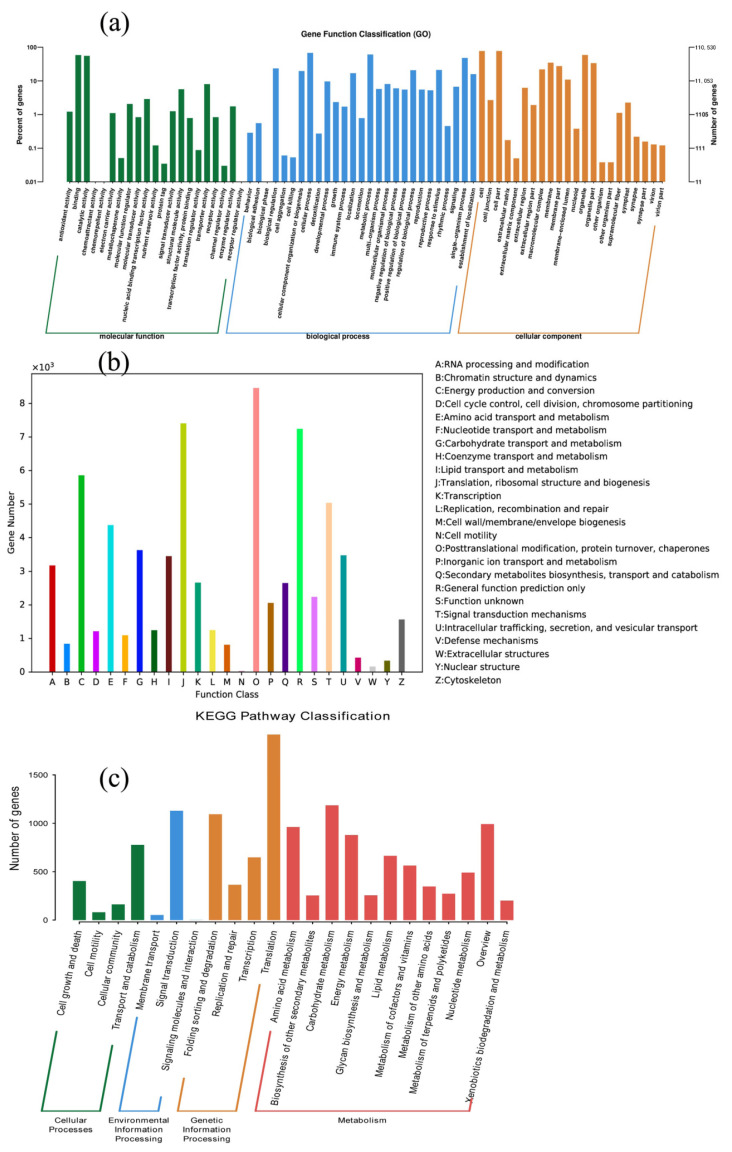
GO (**a**), euKaryotic Ortholog Group (KOG) (**b**) and Kyoto Encyclopedia of Genes and Genomes (KEGG) (**c**) classification of all the identified genes.

In total, the highest annotation ratio was achieved for the GO database (110,530, 44.18%) (Table 2), which represents successful annotation with known proteins. Only 1.84% of the genes were successfully annotated in all the databases; thus, many genes were without annotation. In this study, we focused on the sequence with the highest annotation ratio compared to the GO library to obtain the phase of the gene sequence and functional information for *C. putoensis* and its related species, as long as the gene over 136 K had at least 1 annotation. According to the GO classification (Figure 4a), biological processes (274,614 genes, 36.98%), cellular components (236,419 genes, 31.84%), and molecular functions (231,488 genes, 31.176%) were identified. The KOG classification included 25 functional categories, including posttranslational modification, protein turnover, chaperones (7858 genes, 12.23%), translation, ribosomal structure and biogenesis (6309 genes, 9.82%), and general function prediction only (7041 genes, 10.96%) (Figure 4b). Additionally, the annotated genes were enriched in 23 KEGG pathways (Figure 4c). The top six enriched pathways included translation, carbohydrate metabolism, signal transduction, folding sorting and degradation, overview, and amino acid metabolism.

### 3.4. Identification and Analysis of DEGs in C. putoensis Leaves under NO_2_ Stress

**Figure 5:**
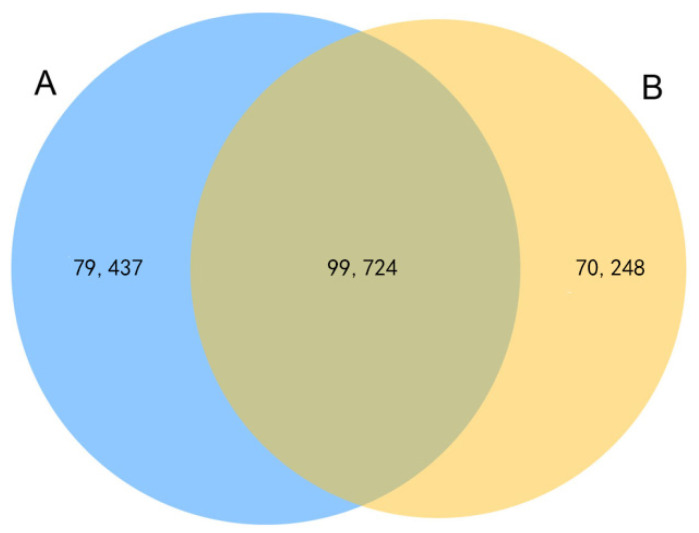
Venn diagram analysis of the expressed genes in two samples (A: Control, B: NO_2_ stressed).

**Figure 6:**
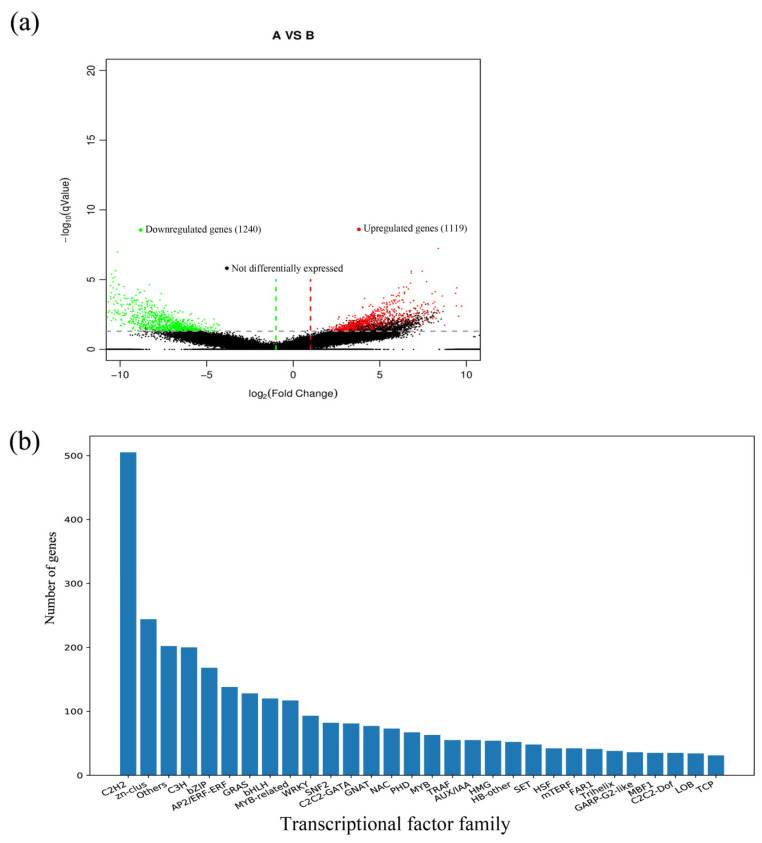
Volcano plots (**a**) of RNA-seq data for an A vs B pairwise comparison; (**b**) the top 30 differentially-regulated TF families were identified among all the genes. A: NO_2_ control. B: NO_2_ stressed. *q*-value, <0.05; fold change, >2.

As in the experimental chambers, all the physical parameters other than the NO_2_ concentration were kept the same; therefore, we presume that the observed results are solely caused by elevated NO_2_. Through the analysis of the CK group and the NO_2_ stress group, the regulatory mechanisms and key genes of *C. putoensis* NO_2_ stress were further explored. To identify DEGs between the two different samples, we analyzed the genes expressed in the two groups; a Venn diagram showed the distribution of specific genes (79,437 and 70,248 expressed genes in the control group (A) and the stressed group (B), respectively) and shared genes (99,724 expressed genes) (Figure 5). Afterwards, pairwise comparisons are performed with FC ≥ 2 and FDR < 0.01 as the standards. In total, the RNA-seq data involved one pairwise comparison, and 2,359 DEGs were ultimately identified, including 1,119 upregulated genes and 1240 downregulated genes (Table 3). The DEGs were annotated using the KOG (877 DEGs, 37.18%), GO (1686 DEGs, 71.47%), KEGG (277 DEGs, 11.74%), and NR (1830 DEGs, 76.6%) databases and the conserved domains database (CDD, 2359 DEGs, 100%) (Table 3). A pairwise comparison of the volcano plots map clearly shows the distribution of upregulated and downregulated genes (Figure 6a). Transcription factors (TFs) are the key components of regulatory systems that control and modulate stress adaptive pathways [22]. In accordance with the highly significant roles of TFs under NO_2_ stress, we analyzed all the genes to identify the top 30 TF families (Figure 6b), which predominantly included *C2H2*, *Zn-clus*, *C3H*, *bZIP*, *AP2/ERF-ERF*, *GRAS*, *bHLH*, *MYB-related, WRKY*, and *NAC*.

**Figure 7:**
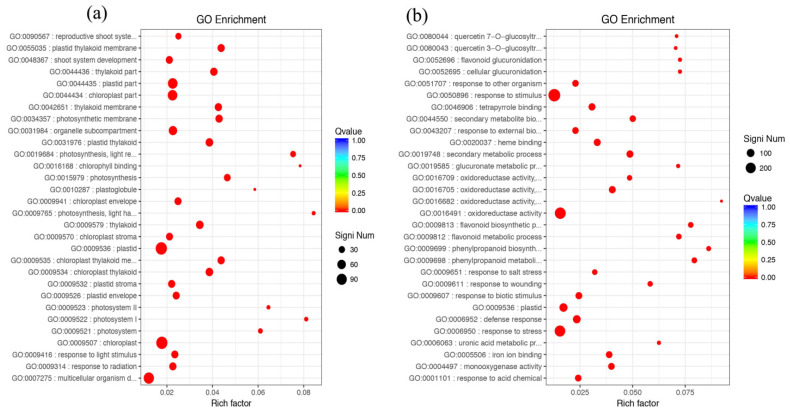
GO enrichment factor analysis of the DEGs. (**a**) Upregulated genes: the top three upregulated genes are involved in multicellular organism development, plastids, and chloroplasts; (**b**) downregulated genes: the downregulated genes predominantly reflected response to stimulus, response to stress, and oxidoreductase activity.

**Figure 8:**
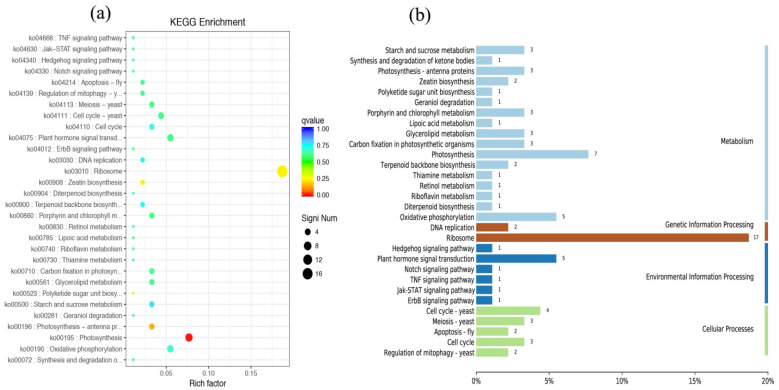
Upregulated genes of KEGG pathway categories (**a**) and enrichment factor analysis (**b**) of the DEGs. The upregulated genes are functionally assigned to 76 biological pathways, and the top upregulated genes are involved in photosynthesis.

**Figure 9:**
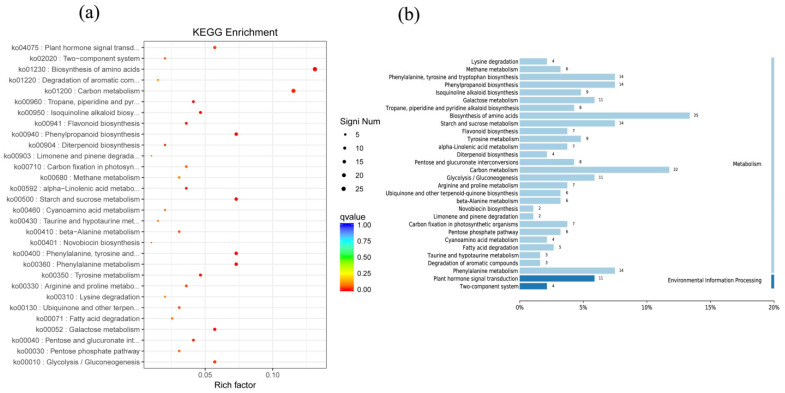
Downregulated genes of KEGG pathway categories (**a**) and enrichment factor analysis (**b**) of the DEGs. The downregulated genes predominantly represent amino acid biosynthesis and carbon metabolism.

**Figure 10:**
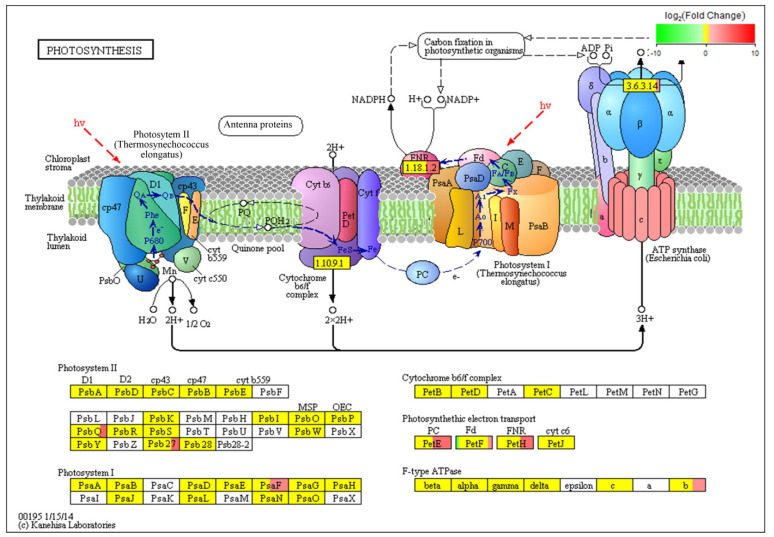
There are 4 genes (*Psb*, *Psa*, *Pet*, and *F-type ATPase a*) involved in photosynthesis in *C. putoensis* under NO_2_ stress. Green represents the downregulated expression of the gene, red represents the upregulated expression of the gene, and yellow indicates no significant difference in gene expression.

The most common enriched pathways were found under GO classification, KEGG pathways, and KOG enrichment. In this study, we analyzed the GO classification of upregulated and downregulated annotated DEGs and selected the 30 with the smallest Q value for a scatter plot of pathway enrichment (Figure 7). The upregulated genes were assigned to 30 biological pathways functionally. The top three upregulated genes were involved in multicellular organism development (GO: 0007275), plastids (GO: 0009536), and chloroplasts (GO: 0009507), and the downregulated genes predominantly reflected response to stimulus (GO: 0050896), response to stress (GO: 0006950), and oxidoreductase activity (GO: 0016491). We also analyzed 91 upregulated and 187 downregulated KEGG pathways annotated with DEGs and chose the 30 with the smallest Q values for scatter plots of the pathway enrichment (Figure 8 and Figure 9). The upregulated genes were functionally assigned to 76 biological pathways; the top upregulated genes were involved in photosynthesis (ko00195) (Figure 8), and the downregulated genes predominantly represented the biosynthesis of amino acids (ko01230) and carbon metabolism (ko01200) (Figure 9). The KEGG pathways showed that the DEGs of the NO_2_-treated group were significantly related to photosynthesis (Figure 10), i.e., four differentially expressed genes were involved in photosynthesis in *C. putoensis* under NO_2_ stress. Combined with the genes classified by GO, which involved plastids and chloroplasts, this finding is consistent with the observed leaf changes in *C. putoensis* under NO_2_ stress, i.e., the color change from green to yellow (shown in Figure 1). This result is also consistent with the change in cell ultrastructure as the chloroplast gradually deforms and more plastid granules appear with increasing NO_2_ stress treatment time, which is a type of abiotic stress (Figure 2).

### 3.5. RT-qPCR Analysis of NO_2_ Stress-related Genes

**Figure 11:**
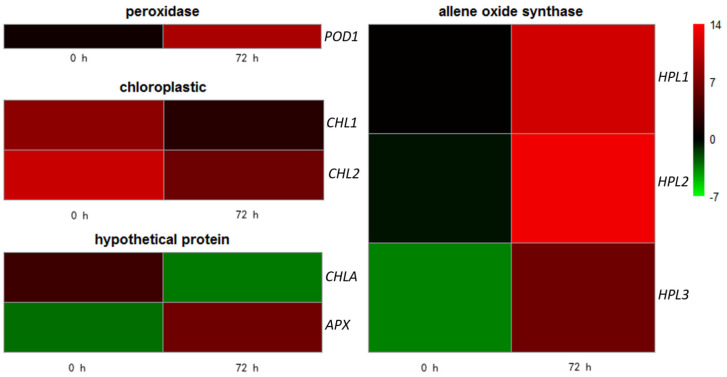
RT-*q*PCR validations of 8 candidate genes involved in NO_2_ stress in *C. putoensis* based on RNA-seq data. Hypothetical protein, chloroplastic, peroxidase, and allene oxide synthase represent different gene types.

**Figure 12:**
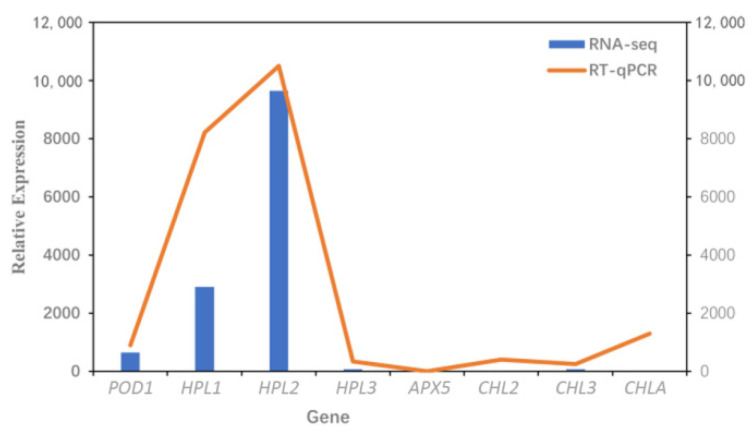
The expression profiles according to RT-qPCR (relative expression) and RNA-seq (FPKM values).

To calculate the accuracy of the RNA-seq, we selected the DEGs with the most significant differences related to NO_2_ stress. We used a functional prediction of annotated genes from the RNA-seq data to identify eight DEGs, namely TRINITY_DN86073_c6_g3 *(peroxidase 12-like*, *POD1*), TRINITY_DN80077_c8_g2 (*allene oxide synthase*, *HPL1*), TRINITY_DN80077_c8_g3 (*allene oxide synthase, HPL2*), TRINITY_DN86773_c3_g1 (*allene oxide synthase, HPL3*), TRINITY_DN81001_c0_g2 (*hypothetical protein CICLE, APX5*), TRINITY_DN86877_c1_g5 (*geranylgeranyl diphosphate reductase, chloroplastic, CHL2*), TRINITY_DN84191_c2_g1 (*chloroplast chlorophyll a/b binding protein, CHL3*), and TRINITY_DN86070_c0_g3 (*hypothetical protein, CHLA*) (Figure 11). RT-qPCR analysis was performed on 8 candidate genes to verify the expression pattern of RNA-seq data (Figure 12). The differential expression profiles of DEGs were consistent between the RNA-seq and RT-qPCR data, except for those of *CHLA*. Although there is a significant difference in the expression profile of one gene, when the RT-qPCR data are compared with the RNA-seq data, there are seven genes that show similar expression profiles. Our study found that *CHL2, CHL3*, and *CHLA* genes showed lower expression levels in the *C. putoensis* leaves upon NO_2_ stress. Strikingly, the selected oxidation family genes *POD1, HPL1*, and *APX5* exhibited higher expression in *C. putoensis* upon NO_2_ stress. These findings seem to suggest that these genes participate in regulating the physiological response of *C. putoensis*.

## 4. Discussion

The results show that gaseous NO_2_ has a significant impact on the ultrastructure of mesophyll cells, i.e., increased translucence in the plastoglobuli, decreased chloroplasts and an increased number of plastoglobuli. Compared with that of the control group, the results are consistent with the gaseous SO_2_ and NO_2_ that cause swelling in the thylakoids and a decrease in the number of grana stacks [31]. The observed changes in the leaf cell structure are similar to those described in Ca-induced plants in the stressed group [30], namely, irregular plastid shape. Part of the reason for these changes may be that NO_2_ changes the semi-permeability of the plastid envelope. NO_2_ can interact directly with lipids, which is probably related to membrane effects [11]. The effects of chemical substances, such as H_2_O_2_ [32], ascorbic acid [33], and Na_2_S [34], have been studied before. However, the effects of natural restoration on plant responses to atmospheric pollution, especially NO_2_, has not been reported before. Our results indicate that natural recovery could be helpful for cell structure recovery and chloroplast morphology. No significant differences were observed between the CK group and the recovered plants, which is consistent with the findings of Souza et al. [35], who found that natural recovery from water stress could lead to the complete recovery of all gas exchange three days after rewatering.

As an important antioxidant enzyme, POD scavenges reactive oxygen species (ROS) [36]. In our experiment, the POD activity increased under NO_2_ stress, indicating that *C. putoensis* plants exhibit substantial ROS-scavenging ability under NO_2_ stress. In tolerant plant species, POD activity is higher, which enables the plants to protect themselves against oxidative stress [37,38]. In *C. putoensis,* it is not known how these changes at the cellular level are regulated at the genetic level. Therefore, we selected the CK group and 72-h NO_2_ treatment group for transcriptome sequencing analysis.

According to the highly significant role of TFs under NO_2_ stress, we analyzed all the genes to identify the top 30 TF families (Figure 6b), which predominantly included *C2H2, Zn-clus, C3H, bZIP, AP2/ERF-ERF, GRAS, bHLH, MYB-related, WRKY*, and *NAC.* These TF families are widely present in a variety of plant species, and they participate in the control of plant development and responses to biotics and biotic stress [39]. Previous research has revealed only the complete chloroplast genome of *C. putoensis* [40]. Our study is the first exploration of these TF families in *C. putoensis* based on transcriptome analysis.

In our experiment, many types of TFs, such as *bZIP, NAC, AP2/ERF,* and *MYB*, are involved in drought stress responses, and *AP2/ERF-ERF* is a large family of TFs in plants. *AP2/ERF-ERF* TFs are identified by the presence of an *AP2* DNA-binding domain composed of 60–70 highly conserved amino acids. *AP2/ERF-ERF* TFs have significant functions in biological processes, including development, reproduction, primary and secondary metabolite biosynthesis, and adaptation to biotic and abiotic stresses [41]. They are primarily activated in response to drought stress [42], heat [43], waterlogging [44], high salinity [45], and osmotic stress [46]; however, this study is the first example of their activation in response to NO_2_ stress. According to the literature, *MYB* TFs play a role in metabolism, cell fate and identity, development, and responses to biotic and abiotic stresses during the plant life cycle [47]. The roles of *WRKY* TFs in plant development, hormone signaling, biotic stress, and abiotic stress have been demonstrated [48]. A transcriptome analysis of *Arabidopsis* roots also indicated the upregulation and downregulation of *WRKY* TFs in response to NO_2_ stress [49]. Plant-specific *NAC* transcription factors have multiple functions, including plant development, defense, and abiotic stress [50]; different plants have different abiotic stress responses to *NAC-TFs* [51]. However, all of the above TFs were determined to have roles in a NO_2_ stress response, and NO_2_ exposure is a type of abiotic stress. 

In our study, several genes that were induced coded for photosynthesis-antenna proteins, and this expression was altered, as shown in Figure 11. The reduction in photosynthesis may also be attributed to degradation and damage in the thylakoid membrane protein-pigment complexes, and possibly also effects on lipids, thereby inducing oxidative stress in stressed plants [52]. As part of a defense mechanism to reduce the oxidative stressed damage, scavenging enzymes such as POD may be activated [53]. In our research, the expression levels of several genes for these enzymes and proteins were modulated. The role of these antioxidants includes altering gene expression to provide a redox buffer and act as a metabolic interface to regulate the optimum induction of adaptive responses [54]. NO_2_ stress has adverse effects on plant growth and productivity; in higher plants, the photosynthesis apparatus is reorganized for acclimation to environmental and metabolic conditions [55]. However, reduced growth under stress is associated with an increase in photosynthesis-related genes, indicating sustained photosynthetic activity under NO_2_ stress [56].

NO_2_ stress leads to enhanced ROS production. In earlier reports, NO_2_ treatment also significantly improved the antioxidant and isozyme activities, including those of superoxide dismutase and POD [57]. These enzymes catalyze the biosynthetic steps of various plant metabolites, and several researchers have demonstrated their role in stress tolerance [58]. Of the 87 *POD genes*, the majority were significantly upregulated after NO_2_ stress, which is consistent with the increase in total POD activity [59]. This is a common response to various oxidative stress factors. Our study identified six differentially expressed transcripts encoding PODs, which are likely involved in the detoxification of ROS in *C. putoensis* under NO_2_ stress and may be potential candidate genes for increasing NO_2_ tolerance.

The results of our study indicate that the effects of NO_2_ exposure on the ultrastructure of the cell structure, POD activity, and morphological changes were directly related to the NO_2_ treatment time; therefore, we speculate that the effects of NO_2_ on plants is partly attributable to the generation and accumulation of N_2_-derived NO_2_^−^ in apoplastic and symplastic spaces. Moreover, with increasing NO_2_ exposure, *C. putoensis* leaves were stressed by a large amount of NO_2_ within a short time, followed by cell membrane damage and chloroplast destruction; this destruction then affected leaf photosynthesis, which altered the expression of genes related to abiotic stress.

Understanding plant stress responses to gaseous pollution is very important for urban greening applications. This study represents the first transcriptome analysis of NO_2_ stress in *C. putoensis*, which is a relatively new field of research, particularly regarding photosynthesis and redox aspects. Therefore, RNA-seq analysis should urgently be conducted to provide further insights into these processes.

## 5. Conclusions

In this study, we recorded the changes in the morphology and anatomy of *C. putoensis* leaves under NO_2_ stress. NO_2_ stress adversely affected the morphology, leaf anatomy, and POD activity. These findings extend our understanding of plant stress responses; they also strongly indicate a need for further RNA-seq analysis. In this study, we used NCBI to compare the transcript with nine databases and obtained its functional annotations. We annotated 2,255 million clean Illumina paired-end RNA-seq reads (clean means to remove the bases with the mass value of reads less than 20 from raw data), and 250,200 unigene sequences were assembled based on the transcriptome data, with an average length of 376.73 bp and an N50 of 381 bp. A comprehensive functional annotation provided functional descriptions for more than 89% of the *C. putoensis* transcripts. Under NO_2_ stress treatment, the plants had 2359 DEGs, among which 1119 exhibited upregulated expression and 1240 exhibited downregulated expression. GO enrichment analysis showed that the DEGs predominantly involved substance metabolism, protein binding, and catalytic activity. The DEGs were typically involved in metabolic pathways and photosynthesis metabolism, which were presented by KEGG analysis. According to the KOG analysis, DEGs were predominantly involved in carbohydrate transport and metabolism, translation, ribosome structure and biogenesis, biosynthesis, and the transport and catabolism of secondary metabolites. According to the KEGG pathway analysis, the expression of photosynthetic genes may be affected by NO_2_ stress. Moreover, GO classification analysis indicated that the chloroplasts, plastids, and stimulus response may be related to NO_2_ stress. Additionally, we found that the expression of POD families experienced dynamic changes during NO_2_ stress treatment. According to the RT-qPCR validation, the *HPL2, HPL1,* and *POD* genes in *C. putoensis* also exhibited high expression under NO_2_ stress. This study provides new insights into the *C. putoensis* processes that occur during NO_2_ stress. Furthermore, the resulting transcriptome data represent an important candidate gene resource for future plant gene structure studies. These data will be very helpful during genome annotation in future genome projects.

## Figures and Tables

**Table 1:** Length and internal length distribution of transcripts and unigenes.

Type	No.	≥500 bp	≥1000 bp	N50	N90	Maximum Length	Minimum Length	Total Length	Average Length	CG Content
Transcript	529,540	124,713	29,088	470	231	7490	201	225,567,341	425.97	40–50%
Unigene	250,200	41,790	9609	381	221	7490	201	94,258,132	376.73	60–70%

**Table 2:** Annotation of unigenes in each database.

Database	Number of Genes	Percentage (%)
CDD	79,760	31.88
KOG	64,226	25.67
NR	94,267	37.68
NT	77,874	31.12
PFAM	51,696	20.66
Swiss-prot	103,389	41.32
TrEMBL	93,882	37.52
GO	110,530	44.18
KEGG	9284	3.71
At least one database	136,276	54.47
All database	4595	1.84

**Table 3:** Annotation of A vs B DEGs in a pairwise comparison.

DEGs	DEG Number	CDD	KOG	GO	KEGG	NR	NT
Upregulated genes	1119	1119	330	690	91	740	597
Downregulated genes	1240	1240	547	996	186	1090	760
Total	2359	2359	877	1686	277	1830	1357

## Data Availability

The data used for the analysis in this study are available within the article and supplementary table.

## Supplementary Materials

The following are available online at https://www.mdpi.com/article/10.3390/genes12050754/s1, Table S1: List of 8 DEG primers used for RT-qPCR. Raw sequence data are available at http://www.ncbi.nlm.nih.gov/bioproject/717124 (accessed on 10 May 2021), with BioSample accession number of SAMN19071592, SAMN19071593 and SRA accession number of PRJNA717124.

## References

[1] Rahmat M., Maulina W., Rustami E., Azis M., Budiarti D., Seminar K., Yuwono A., Alatas H. (2013). Performance in real condition of photonic crystal sensor based NO_2_ gas monitoring system. Atmos. Environ..

[2] Sasakawa H., Yoneyama T. (1979). Transformation of atmospheric NO_2_ absorbed in spinach leaves. Plant Cell Physiol..

[3] Bermejo-Orduna R., McBride J., Shiraishi K., Elustondo D., Lasheras E., Santamaría J. (2014). Biomonitoring of traffic-related nitrogen pollution using *Letharia vulpina* (L.) Hue in the Sierra Nevada, California. Sci. Total Environ..

[4] Lu M., Li Y.J., Lu J.P. (2002). The study of greening trees on the atmospheric pollutant absorption ability. J. Urban. Environ. Urban. Ecol..

[5] Stulen I., Pérez-Soba M., de Kok L.J., van der Eerden L. (1998). Impact of gaseous nitrogen deposition on plant functioning. New Phytol..

[6] Morikawa H., Takahashi M., Sakamoto A., Matsubara T., Arimura G.-I., Kawamura Y., Fukunaga K., Fujita K., Sakurai N., Hirata T. (2004). Formation of unidentified nitrogen in plants: An implication for a novel nitrogen metabolism. Planta.

[7] Nussbaum S., von Ballmoos P., Gfeller H., Schlunegger U.P., Fuhrer J., Rhodes D., Brunold C., Ballmoos P. (1993). Incorporation of atmospheric 15NO_2_-nitrogen into free amino acids by Norway spruce *Picea abies* (L.) Karst. Oecologia.

[8] Weber P., Nussbaum S., Fuhrer J., Gfeller H., Schlunegger U.P., Brunold C., Rennenberg H. (2006). Uptake of atmospheric 15NO_2_ and its incorporation into free amino acids in wheat (*Triticum aestivum* L.). Physiol. Plant..

[9] Beneoict H.M., Breen W.H. (1995). The use of weeds as a means of evaluating vegetation damage caused by air pollution. Proc. Nat. Ah. Polhtt. Syrup.

[10] Middleton J.T., Darley E.F., Brewer R.F. (1958). Damage to Vegetation from Polluted Atmospheres. J. Air Pollut. Control. Assoc..

[11] Mayer D., Mithofer A. (2018). Short-term exposure to nitrogen dioxide provides Bbasal pathogen resistance. Plant. Physiol..

[12] Morikawa H., Higaki A., Nohno M., Takahashi M., Kamada M., Nakata M., Toyohara G., Okamura Y., Matsui K., Kitani S. (1998). More than a 600-fold variation in nitrogen dioxide assimilation among 217 plant taxa. Plant. Cell Environ..

[13] Yang J., Caia L., Liua D., Chen G., Gratzfeld J. (2020). China’s conservation program on Plant Species with Extremely Small Pop-ulations (PSESP): Progress and perspectives. Biol. Conserv..

[14] Ma Q.F. (2016). The Adjustment of Endogenous Salicylic Acid on NO_2_ Stress in Arabidopsis.

[15] Zhong T.L., Li G.Y., Shi B.L. (2009). Comparison of gas exchange and chlorophyll fluorescence parameters in three endangered species of Zhejiang Province. J. Shanghai Jiaotong Univ. Agric. Sci..

[16] Sheng Q.Q., Zhu Z.L. (2018). Photosynthetic Capacity, Stomatal Behavior and Chloroplast Ultrastructure in Leaves of the Endan-gered Plant *Carpinus putoensis* W.C.Cheng during Gaseous NO_2_ Exposure and after Recovery. Forests.

[17] Allen R.G., Jensen M.E., Wright J.L., Burman R.D. (1989). Operational estimates of evapotranspiration. Agron. J..

[18] Asada K. (1994). Production and Action Oxygen in Photosynthetic Tissue.

[19] Wu Y.X., Andreas V.T. (2002). Impact of fungicides on active oxygen species and antioxidant enzymes in spring barley (*Hordeum vulgare* L.) exposed to ozone. Environ. Pollut..

[20] Shimazaki K.-I., Yu S.-W., Sakaki T., Tanaka K. (1992). Differences between Spinach and Kidney Bean Plants in Terms of Sensitivity to Fumigation with NO_2_. Plant Cell Physiol..

[21] Wang L., Stegemann J.P. (2010). Extraction of high quality RNA from polysaccharide matrices using cetlytrimethylammonium bromide. Biomaterials.

[22] Wang L., Wang S., Li W. (2012). RSeQC: Quality control of RNA-seq experiments. Bioinformatics.

[23] Okonechnikov K., Conesa A., García-Alcalde F. (2016). Qualimap 2: Advanced multi-sample quality control for high-throughput sequencing data. Bioinformatics.

[24] Patro R., Duggal G., Love M.I., Irizarry R.A., Kingsford C. (2017). Salmon provides fast and bias-aware quantification of transcript expression. Nat. Methods.

[25] Florea L., Song L., Salzberg S.L. (2013). Thousands of exon skipping events differentiate among splicing patterns in sixteen human tissues. Research.

[26] Ogata H., Goto S., Sato K., Fujibuchi W., Bono H., Kanehisa M. (1999). KEGG: Kyoto Encyclopedia of Genes and Genomes. Nucleic Acids Res..

[27] Finn R.D., Coggill P., Eberhardt R.Y., Eddy S.R., Mistry J., Mitchell A.L., Potter S.C., Punta M., Qureshi M., Sangrador-Vegas A. (2016). The Pfam protein families database: Towards a more sustainable future. Nucleic Acids Res..

[28] Szklarczyk D., Franceschini A., Wyder S., Forslund K., Heller D., Huerta-Cepas J., Simonovic M., Roth A., Santos A., Tsafou K.P. (2015). STRING v10: Protein–protein interaction networks, integrated over the tree of life. Nucleic Acids Res..

[29] Yates A., Akanni W., Amode M.R. (2016). Ensembl 2016 Nucleic Acids Research. Oxford Acad..

[30] Oulhen N., Foster S., Wray G., Wessel G. (2019). Identifying Gene Expression from Single Cells to Single Genes. Methods Cell Biol..

[31] Schiffgens-Gruber A., Lütz C. (1992). Ultrastructure of mesophyll cell chloroplasts of spruce needles exposed to O_3_, SO_2_ and NO_2_ alone and in combination. Environ. Exp. Bot..

[32] Wu G., Shortt B.J., Lawrence E.B., Levine E.B., Fitzsimmons K.C., Shah D.M. (1995). Disease resistance conferred by expression of a gene encoding H_2_O_2_-generating glucose oxidase in transgenic potato plants. Plant. Cell.

[33] Shalata A., Neumann P.M. (2001). Exogenous ascorbic acid (vitamin C) increases resistance to salt stress and reduces lipid peroxi-dation. J. Experiment. Bot..

[34] Hu Y., Bellaloui N., Sun G., Tigabu M., Wang J. (2014). Exogenous sodium sulfide improves morphological and physiological responses of a hybrid Populus species to nitrogen dioxide. J. Plant. Physiol..

[35] Souza R.P., Machado E.C., Silva J.A.B., Lagoa A.M.M.A., Silveira J.A.G. (2004). Photosynthetic gas exchange, chlorophyll fluo-rescence and some associated metabolic changes in cowpea (*Vigna unguiculata*) during water stress and recovery. Environ. Experiment. Bot..

[36] Liu B., Kang C., Wang X., Bao G. (2016). Physiological and morphological responses of *Leymus chinensis* to saline-alkali stress. Grassl. Sci..

[37] Scalet M., Federice R., Guido M.C., Manes F. (1995). Peroxidase activity and polyamine changes in response to ozone and simulated acid rain in Aleppo pine needles. Environ. Experiment. Bot..

[38] Ren Y., Wang W., He J., Zhang L., Wei Y., Yang M. (2020). Nitric oxide alleviates salt stress in seed germination and early seedling growth of pakchoi (*Brassica chinensis* L.) by enhancing physiological and biochemical parameters. Ecotoxicol. Environ. Saf..

[39] Zhao P., Hou S., Guo X., Jia J., Yang W., Liu Z., Chen S., Li X., Qi D., Liu G. (2019). A MYB-related transcription factor from sheepgrass, LcMYB2, promotes seed germination and root growth under drought stress. BMC Plant. Biol..

[40] Feng S., Xie X.Y., Wang M.C., Yang Y.Z. (2017). Characterization of the complete chloroplast genome of *Carpinus putoensis*. Conserv. Genet. Resour..

[41] Licausi F., Kosmacz M., Weits D., Giuntoli B., Giorgi F.M., Voesenek L.A.C.J., Perata P., Van Dongen J.T. (2011). Oxygen sensing in plants is mediated by an N-end rule pathway for protein destabilization. Nat. Cell Biol..

[42] Golldack D., Lüking I., Yang O. (2011). Plant tolerance to drought and salinity: Stress regulating transcription factors and their functional significance in the cellular transcriptional network. Plant. Cell Rep..

[43] Sakuma Y., Maruyama K., Qin F., Osakabe Y., Shinozaki K., Yamaguchi-Shinozaki K. (2006). Dual function of an Arabidopsis transcription factor DREB2A in water-stress-responsive and heat-stress-responsive gene expression. Proc. Natl. Acad. Sci. USA.

[44] Hinz M., Wilson I.W., Yang J., Buerstenbinder K., Llewellyn D., Dennis E.S., Sauter M., Dolferus R. (2010). Arabidopsis RAP2.2: An Ethylene Response Transcription Factor That Is Important for Hypoxia Survival. Plant. Physiol..

[45] Abogadallah G.M., Nada R.M., Malinowski R., Quick P. (2011). Overexpression of HARDY, an AP2/ERF gene from Arabidopsis, improves drought and salt tolerance by reducing transpiration and sodium uptake in transgenic *Trifolium alexandrinum* L. Planta.

[46] Zhu Q., Zhang J., Gao X., Tong J., Xiao L., Li W., Zhang H. (2010). The Arabidopsis AP2/ERF transcription factor RAP2.6 participates in ABA, salt and osmotic stress responses. Gene.

[47] Dubos C., Stracke R., Grotewold E., Weisshaar B., Martin C., Lepiniec L. (2010). MYB transcription factors in Arabidopsis. Trends Plant. Sci..

[48] Li H.-L., Qu L., Guo D., Wang Y., Zhu J.-H., Peng S.-Q. (2020). Histone deacetylase interacts with a WRKY transcription factor to regulate the expression of the small rubber particle protein gene from Hevea brasiliensis. Ind. Crop. Prod..

[49] Burner J., West B., Mayer P.M. (2019). What do we expect from the dissociation of ionized nitro-substituted polycyclic aromatic hydrocarbons in the interstellar medium?. Int. J. Mass Spectrom..

[50] Olsen A.N., Ernst H.A., Leggio L.L., Skriver K. (2005). NAC transcription factors: Structurally distinct, functionally diverse. Trends Plant. Sci..

[51] Wang M., Zou Z., Li Q., Sun K., Chen X. (2017). The CsHSP17.2 molecular chaperone is essential for thermos tolerance in Camellia sinensis. Sci. Rep..

[52] Shah J.M., Bukhari S.A.H., Zeng J.B., Quan X.Y., Ali E., Muhammad N., Zhang G.-P. (2017). Nitrogen (N) metabolism related enzyme activities, cell ul-trastructure and nutrient contents as affected by N level and barley genotype. J. Integrat. Agric..

[53] Mukherjee A., Agrawal S.B., Agrawal M. (2020). Responses of tropical tree species to urban air pollutants: ROS/RNS formation and scavenging. Sci. Total Environ..

[54] Miller G., Suzuki N., Ciftci-Yilmaz S., Mittler R. (2010). Reactive oxygen species homeostasis and signalling during drought and salinity stresses. Plant Cell Environ..

[55] Kargul J., Barber J. (2008). Photosynthetic acclimation: Structural reorganization of light harvesting antenna–role of redox-dependent phosphorylation of major and minor chlorophyll a/b binding proteins. FEBS J..

[56] Bahl A., Kahl G. (1995). Air pollutant stress changes the steady-state transcript levels of three photosynthesis genes. Environ. Pollut..

[57] Xu Q., Zhou B., Ma C., Xu X., Xu J., Jiang Y., Liu C., Li G., Herbert S.J., Hao L. (2010). Salicylic Acid-Altering Arabidopsis Mutants Response to NO_2_ Exposure. Bull. Environ. Contam. Toxicol..

[58] Khare T., Kumar V., Kishor P.B.K. (2015). Na+ and Cl− ions show additive effects under NaCl stress on induction of oxidative stress and the responsive anti-oxidative defense in rice. Protoplasma.

[59] An J.P., Li R., Qu F.J., You C.X., Wang X.F. (2017). Ectopic expression of an apple cytochrome P450 gene MdCYPM1 negatively regulates plant photo morphogenesis and stress response in Arabidopsis. Biochem. Biophys. Res. Commun..

