# Supplementary material for: Comprehensive Transcriptome Analysis of Rare Carpinus putoensis Plants under NO2 stress
Source: Genes (Basel). 2021 May 17;12(5):754. doi: 10.3390/genes12050754 (PMC8156095; doi:10.3390/genes12050754)
Supplement: Supplementary file 1 [file genes-12-00754-s001.zip › genes-1110121 supplementary.pdf]

Supplementary Table S1. List of 8 DEG primers used for RT-qPCR

Gene id	Primer
TRINITY_DN86073_c6_g3 (peroxidase 12-lik, POD1)	Upstream: TATGTTTGTCCCACCGAAGCG<br>Downstream: CCTTTGTCCTGCTATCCGTGT
TRINITY_DN80077_c8_g2 (allene oxide synthase, HPL1)	Upstream: CGGCACAAACTCTTCGGCTTT<br>Downstream: TAGAACCGCCAGTACCGCTCCA
TRINITY_DN80077_c8_g3 (Allene oxide synthase, HPL2)	Upstream: AAGTCCCCGTGAAAAGGTCT<br>Downstream: GCCCTTTCATAGCCTCCGAT
TRINITY_DN86773_c3_g1 (Allene oxide synthase, HPL3)	Upstream: CCTTCATCTGTGACAACGGTCCT<br>Downstream: AAGACGACGTTTCCCGCTCTG
TRINITY_DN81001_c0_g2(hypothetical protein CICLE, APX5)	Upstream: TACTTTGACACCGAGCACACC<br>Downstream: GCCTCCCTTAACTCAGATGGGTA
TRINITY_DN86877_c1_g5 (geranylgeranyl diphosphate reductase, chloroplast dimer, CHL2)	Upstream: ATCATACGAGTTGAGGCACACC<br>Downstream: ATTCTCCGACCCTTCAACCAC
TRINITY_DN84191_c2_g1 (chloroplast chlorophyll a/b binding protein, CHL3)	Upstream: GCAATACGGTAGCCCTCGACT<br>Downstream: ACATTTGCCAGGAACCGTGA
TRINITY_DN86070_c0_g3 (hypothetical protein, CHLA)	Upstream: CTGGCCCGGCATCCAATCG<br>Downstream: TTCGTCAGTATCGGTAGCGGTCT
